# Medical Notes and Reflections

**Published:** 1839

**Authors:** 


MEDICAL NOTES AND REFLECTIONS. By *Henry Holland,* M.D. F.R.S. &c. Fellow of the Royal College of Physicians, and Physician Extraordinary to the Queen. Octavo, pp. 628. London: Longman and Co. 1839.

Of the thirty-five chapters which compose this work, we have noticed eleven more or less circumstantially. Works of this kind are rare now-a-days. They have been superseded by researches of a more anatomical character, by observations of a statistical nature, and by inquiries which have aimed at giving exactness to the science of medicine.

It would be idle to dwell on the benefits which have accrued from this modern plan of proceeding. They have stripped physic of much of the empiricism and of more of the dogmatism of former days. The revolutions which then disgraced it cannot again convulse it, for its basis is found in the positive truths of pathology. Those truths may be extended, curtailed, or modified with the progress of discovery, but the alterations will be limited by reasonable bounds, and, unlike the old changes from sect to sect, and from one absurd opinion to its contrary, they will not throw an air of ridicule upon the art.

But, valuable as this spirit of investigation has proved, and philosophical as it is, it must be conjoined with a rational empiricism. The properties of drugs and their effects upon disease are only to be determined by experiment and observation. Such experiments and such observations are as requisite, to render medicine practical, as morbid anatomy is to make it exact. Each is indispensable, and it is but rarely that the same individual is equally fitted to engage in each.

We need hardly say that Dr. Holland's book offers us the results of his personal experience. His opportunities have been considerable, and his mind has been cultivated with sufficient assiduity and success, to enable him to observe with acuteness, and to report his observations with accuracy and with clearness. Such books are, perhaps, less numerous than they should be. Men of large experience alone can produce them, and men of large experience have seldom leisure or inclination for the purpose.

It signifies so little what chapter we take first, what last, of Dr. Holland's book, that convenience or caprice will regulate our selection. We shall touch most lightly or not at all upon those, which have no particular bearing upon practice.

### On the Influence of Weather in relation to Disease.

Such is the title of the twenty-eighth chapter. Dr. Holland very justly remarks, that—amidst all the opinions and phrases current on this subject, it is singular how little real knowledge has been gained, or applied to practice. The difficulties of the inquiry are great, both from the complexity of the agents concerned, and from that of the organs and functions acted upon. Yet with the certainty we have of the extent, variety, and unceasing nature of this influence, it is manifestly a field of research, where patient observations and averages cannot fail of affording the most valuable results.

Dr. Holland goes on to observe that the subject, taken in its whole extent, admits of being distributed into several separate objects of inquiry.

First, there is the history of the diseases of particular climates, on which a good deal has been done—then we must consider the climate of particular places, and their relative fitness as residences for invalids—then, the relation of the several states and changes of atmosphere to the various functions of the body; a wide topic, abounding in curious results, and these of equal interest to physiology and pathology.

Dr. Holland's own observations refer, however, more especially to the influence of certain states of air or weather in producing morbid conditions of the body; either in their immediate effect, or indirectly by evolving or giving greater activity to other causes.

After a recommendation to all practitioners, to endeavour to connect, so far as that can be properly done, the prevalence, type, and change of disease on a single spot, with corresponding conditions in the atmosphere around it, Dr. Holland proceeds:—

"But besides the recognized diseases of certain seasons, there are others not in general so considered, which nevertheless are frequent enough, at particular times, to make it probable that atmospheric influence is concerned. My notes furnish me with many instances of this kind; some of them, indeed, liable to question, as being possibly the result of casual or unknown causes; yet deserving notice in the suggestions they afford for future observation. It cannot be doubted that affections of the head, depending on disturbed balance of circulation through the brain, do at some periods much exceed the common average of these cases. The same remark I would extend, on my experience, to various neuralgic disorders; including under these some affections, usually termed rheumatic, which are thus, however, most correctly classed. Lumbago and sciatica are undoubtedly more prevalent in certain states of atmosphere: and we may recognise a similar effect in the familiar instance of pain renewed in old sprains, or in joints affected by former inflammation. Further, though more doubtfully, I have found reason to think that attacks of gout, in patients having this habit of body, are more common than usual at certain periods, without other cause to which this can reasonably be assigned.

With respect, again, to disorders of the internal membranes; while many of these are noted as prevailing at particular seasons, there are others not thus described, yet which observation shows to depend greatly on external causes, giving them frequently an epidemic character. Every physician must have noticed a class of bowel disorders, almost specific in kind, which prevail extensively at one time; while, at another, there is the tendency, not less marked, to different forms of cynanche, or to erythematous inflammations of the fauces, palate, and gums. Erysipelas evidently prevails more generally and severely under particular states of weather, and these not connected with any regular occurrence or character of seasons. The infantile fever, however it be classed in our nosology, is clearly the subject of external influences, to the same extent, and possibly in the same way, as the exanthematous disorders. And with respect to gastric fevers in general, whatever name they assume, we have evidence of their becoming epidemic under certain continued states of weather; and apparently alike at different periods of their occurrence.

Hooping-cough may be further mentioned, as obviously partaking in the same liability. The different affections of the membrane of the air-passages,—catarrh, croup, asthma, and bronchitis,—though not all depending on this cause solely or equally, yet are greatly under its influence; and rendered more frequent or severe as particular conditions of atmosphere are present, or prolonged in duration. Even brochocele, a disorder of which it is so difficult to render any plausible account, seems to afford another example of this influence.* Other glandular affections are much more frequently and certainly submitted to it." 460.

Setting aside accidental miasmata in the air, any inquiry, continues our author, respecting the influence of weather in the production or modification of disease, must include four principal objects:—*first*, the temperature of the air;—*secondly*, its hygrometrical state;—*thirdly*, its weight;—and *fourthly*, its electrical state and changes. The agency of winds may obviously be referred in great part to one or other of these conditions. That of light, though the facts are sufficient fully to prove its existence, is too subtle to be well defined in the present state of our knowledge.

It is impossible to follow Dr. Holland in detail. We must select such passages or such ideas as appear to court particular attention. Amongst these we observe some remarks on the application of cold in fevers, which though not novel are, we think, very true.

"Almost may it be taken as a rule, that wherever there is a hot and dry skin, cold in one degree or other may safely and expediently be applied to change its state. The benefit of simple abstraction of heat is great in such cases; and the fact is not sufficiently adverted to, which I have often put to thermometrical test, of the extent to which this influence is diffused beyond the surface to which the cold is immediately applied. There is no real risk here to countervail the good gained. We are sedulous in providing for and varying the application of heat to the body; while, from one cause of alarm or another, little provision is made for the opposite remedy, though not less capable of being actively and beneficially employed." 469.

As Dr. Holland observes, the facts and recommendations of Dr. Currie have either not sunk deep into our minds, or popular prejudice has struggled very successfully against them.

---

* "One or two instances are known to me, where the ordinary prevalence of bronchocele in a particular locality has been suddenly augmented by many new cases; sometimes numerously in the same family. The cynanche parotidæa is much more familiar as an epidemic at certain periods, in which effect weather may also be concerned; though it is more probable, from the specific and infectious nature of the complaint, that this is only partially the case."

Dr. Holland concludes his observations on the effects of temperature, with a hint or two of some importance. In practice, he says, and for a rule in the habits of life, regard is not sufficiently paid to the different power which different individuals possess, of generating animal heat. This function, whether depending on changes in the blood and manner of circulation, or more directly on the nervous system, is as various in its power and exercise as any other of the body, and requires to be dealt with as such. Each age, too, has its changes in this respect, as well as every condition of health; and precautions founded on them cannot expediently be neglected, provided they are not so minute as to interfere with other parts of the economy of life, equally essential to the welfare of the whole. We have been struck with a piece of the most absurd and pernicious cruelty to children, which appears at present to be quite the mode. The experiments of Dr. Edwards have shewn conclusively that young animals possess a power of generating caloric, very inferior to that enjoyed by their seniors. In despite of this fact, in contempt of the laws of nature, and in utter defiance of common decency and common feeling, children have now their legs and bottoms bared, and are sent into the streets even in cold weather, with clothing which would qualify them for the ballet at the opera-house. If the fathers and mothers of these poor little urchins were served in the same way, they would get no more than their deserts, and their chilblains, rheumatisms, and pleurisies would bring them to their senses pretty speedily.

There are some reflections on alterations in the weight of the atmosphere which appear to us to be very just.

Notable effects, says our author, may occur, when the changes in the weight of the air are frequent, sudden, and considerable, even within the ordinary range of atmospheric variation. Regarding merely the average pressure upon the whole body, it is to be supposed that any very sudden fluctuation, to the amount perhaps of one thirtieth, may produce temporary changes in the balance of circulation between external and internal parts, of much moment to the latter. And these are particularly to be looked for when there is individual liability to certain diseases, or close approach to them at the time. Dr. Holland continues :—

"This observation, as I have already stated, appears especially to apply to affections of the brain. I have made note of two or three periods, since I began practice, during which there has been a more than wonted frequency of apoplectic or paralytic seizures within my immediate knowledge; so marked as to make it difficult to attribute the fact to mere casualty, notwithstanding the many circumstances which tend to invalidate such results when not verified by large averages. The same fact, observed by others, has generally been attributed to external heat alone. But allowing what has already been assigned to this cause, the particular character of the weather at these times will scarcely support the inference; nor has the result in question been equally apparent even under higher degrees of atmospheric temperature. While, on the other hand, I have observed at these periods frequent and rapid changes in the barometer, often with great depression of its level; and have noticed at the same time the very common occurrence of lesser affections of the head,—vague and uneasy sensations, oppression, vertigo, and what may be termed a feeling of want of proper balance to the frame—all indicating some cause present which tends more or less to disturb the equality of circulation through this organ.

In fact, the ordinary phrases of heaviness and lightness of air (however misplaced or even inverted their use) prove the general consciousness of these changes in their slighter influence on the body. It may be difficult to say through what organ or function this feeling is chiefly conveyed; but probably it is a compound effect of the changes in circulation, in which the sensorium, the lungs, and the muscular system, all participate. Even the organs of digestion seem to be affected, directly or indirectly, by the same causes. Without referring to the doubtful instance of vomiting produced in highly rarified air, I think I have observed frequent disturbance both in the sensations and functions of the alimentary canal, under any rapidly diminished weight of the atmosphere, or where its changes were more frequent than usual. I have remarked in the preceding chapter on the indications of disturbance to sleep from the same cause." 482.

We believe that there is much truth in this, and practical men would do well to look to these things rather more closely than they have done. Who has not observed the effect of hygrometric and barometric changes on the head and on the liver? Bilious attacks, as they are denominated, occur as frequently in consequence of changes of weather, as of imprudences in diet. When one perceives the effect of atmospheric alterations on the circulation, can we wonder at the implication of the brain or of the alimentary apparatus?

After many other considerations, Dr. Holland concludes that,—On a general view of the circumstances stated, there is reason to conclude that the influence of the different degrees of atmospheric pressure in disturbing the bodily functions and general health, is rather derived from the frequency of fluctuations, than from any state long continued, either above or below the average standard;—that, of the two conditions, suddenly incurred in any extreme degree, the human frame is better capable of withstanding a rarefied than a condensed atmosphere;—and that, in every case, the previous health, and proneness to disorder in particular organs, are greatly concerned in determining the results on the body.

Dr. Holland touches, and not very slightly, on the effects of an electrical condition of atmosphere. He must be sceptical, indeed, who can refuse assent to the belief that such effects on the human body are important. Our own feelings, universal experience, convince us that they are. But their amount and nature are certainly anything but understood at present. This will be our excuse for not alluding more particularly to our author's remarks. They bear the usual impress of his research and observation, but they are too indeterminate to give us confidence to cite them. The only passage to which we would direct attention, is the following:—

"In relation to future inquiry on this subject, the application of electricity as a medicinal agent may best be viewed in the three following ways: first, in its action on the nervous system; secondly, on the blood; and thirdly, on particular organs under disease. The first two topics are the most important; and all that has been stated above tends to show that we may look to these as a basis of future discovery. Many results already obtained (as those recently published by Dr. Addison, in relation to chorea, and the successful experiment of Matteucci in tetanus), make it probable that the use of electricity in nervous and spasmodic disorders, even when depending on the sensorium, may be greatly extended; and though its action on morbid states of the blood seems much less within our reach, seeing the multiform conditions of this fluid, and the practical difficulties of application, yet is the object one well meriting diligent research." 498.

### ON THE PRESENT QUESTIONS REGARDING VACCINATION.

Dr. Holland makes some judicious observations on the existing state and the future prospects of vaccination. His argument, or rather his statement of the arguments, need not be entered on. We shall only cite one or two of his opinions, as the sentiments of a man of his experience and character are naturally sought and valued.

He observes, in reference to the duration of the protective influence of vaccination, a point so much agitated, and determined by many with such ridiculous precision, that—there is cause to believe that in some instances the protection has been extended over a period of thirty-five years; but the average duration of that which is completely effective must be rated far below this mark. It has been a question naturally occurring, whether its diminished influence is in any general proportion to the interval of time succeeding vaccination. The documents obtained in England and elsewhere, as well as the results of re-vaccination, favour the idea that such proportion exists; probably not with any exact uniformity of rate, but determined in part by causes intermediately affecting the body. These documents concur in showing a maximum in the number of small-pox cases after vaccination, from the age of fifteen to twenty-five;—few before ten years of age;—and a diminishing number after twenty-five, as might be inferred from other considerations. This at present seems the utmost extent of our knowledge on the subject.

It must certainly be deemed in the present condition of our information, a very vague, if not a precipitate measure, to fix on any term of years as the period of protection. But if statistics can do anything for us, they are likely to do this—and to statistics alone we must look for the information. In the course of the next ten years we may anticipate a large body of valuable facts for our instruction.

Dr. Holland, like a man of sense, takes a practical and favourable view of vaccination. That it is not a certain preventive, is true; but it is in the bulk of cases a preventive, and, where it does not prevent small-pox, it almost always mitigates it.

Pitch the merits of vaccination as low as with any sort of fairness we can, and it remains an unquestionable benefit—such a benefit as was never before conferred on the physical nature of man.

Of the means for attaining a higher security on the part of vaccination, Dr. Holland naturally enumerates:—First, the obtaining fresh and genuine vaccine lymph from its source. Secondly, re-vaccination at certain periods, and the determination of the times best fitted for this. Thirdly, improvement in the methods and tests of perfect vaccination. And, fourthly, the influence, moral or otherwise, which is needful with the world, to obtain a more complete and exclusive use of this preventive remedy. Of any *but* moral means under the latter head, we own that we are far from sanguine. Stringent measures sin against the spirit of the age, and the personal liberty which is claimed by the citizens of all free states. It is true that, in instances of this kind, an infringement of such liberty would be for the good of those who are coerced. But that coercion implies power, which, though exercised to-day for good, may to-morrow be employed for evil; and, unfortunately, the experience of men has taught them that the evil will be inflicted oftener than the good. Despotism has its advantages, but liberty has greater; and if the latter brings some mischiefs in her train, she but shares the imperfections of all earthly things.

Dr. Holland is an advocate for replacing the vaccine matter, now in circulation. But, as he justly states, unhappily, the means of attaining this object have not corresponded with its urgency, or with the common impression prevailing on the subject. In England, as in other parts of the world, there has been found an unexpected difficulty in recovering the genuine vaccine lymph. The disease is not only rarer among cows than it was presumed to be, but blended in them with other pustules and vesicular diseases; several of these affecting the human subject with a spurious pock; others incommunicable; but all adding to the embarrassment of the research. We need scarcely add, that Mr. Estlin has succeeded in the most gratifying manner, in obtaining it.

Alluding to re-vaccination, Dr. Holland throws out these suggestions:—

"It becomes of consequence to define the best period for a practice unequivocally desirable at one time or another. And, reviewing all that has yet been learnt on the subject, I am led to believe that where there is fair proof of the original vaccination being perfect in kind, the period best to be selected for re-petition is that from ten to twelve years of age;—a time anterior to the more explicit changes of puberty, and preceding the age at which the liability to small-pox is shewn by averages to be again most frequently renewed. Future facts may modify or alter this view; but meanwhile it appears to be sanctioned by the evidence we possess, and is liable to no risk of inflicting injury, even if hereafter found to be less expedient than some other yet undetermined. And it may be added, that re-vaccination, securely performed at the period named, might possibly render any further repetition needless; such inference being drawn from the tables in our hands, and from the general fact of liability to small-pox diminishing as life proceeds. It must be admitted, however, as still more open to future corrections than the former, and can only indeed be determined by time." 411.

All things considered, Dr. Holland takes the age of two or three months to be the fittest for the primary vaccination—avoiding, as we do, thereby on the one hand, the rashes and bowel disorders, which often occur in the first weeks of life;—on the other, the period of teething and the various febrile irritations which attend it;—while at the same time not unduly exposing the child to the intermediate risk of small-pox.

We quite agree with our author that there ought to be no mystification with the public. The case should be stated as it is. We should *not* say that vaccination is a certain preventive of small-pox. But we *should* say, that it is a very likely preventive, and that with the precaution of occasional re-vaccination, it will probably be an effectual one. The evidence in favour of vaccination is as strong as that in favour of almost every thing which men generally deem eligible in life—a strong balance of chances on its side. Dr. Holland recommends the following mode of putting the case to a parent:—

"Were I to suggest any manner of reasoning, most generally effective for these objects, and best according with the truth of facts, it would be some such as the following:—'Your child is at this moment liable to a dangerous and loathsome disease. If you inoculate him for small-pox, you give the disease in a milder form indeed, but always distressing, sometimes fatal, and not absolutely certain as a safeguard against recurrence. If you vaccinate, you afford him, without pain or risk of any kind, protection probably complete for a period of years: capable of being readily renewed; and, when failing of completeness, greatly mitigating the severity of the disorder.'

To some persons the motive may be added, that by inoculating for the small-pox they become a party to keeping this poison still in the world. But practically it may be found better to limit the reasoning as stated above. And the argument for re-vaccination, as the facts now stand, may be enforced in the same sense. 'Your child has had perfect protection for a certain period of life. There is cause from experience to presume that this protection is now drawing to an end, or perhaps is altogether lost. By re-vaccination you renew it, if renewal be required, without risk, suffering, or the confinement of a single hour.' " 416.

From a chapter,

### On Sleep,

We only extract the following observations:—

"I have found that any very sudden and considerable fall of the barometer produces in many persons a sort of lassitude and drowsiness, followed by restless and uneasy sleep, or frequently a state of laborious dreaming. It may be doubted whether this influence depends on changes in the balance of circulation thus produced, or on alterations in the electrical state of the air concurring with its change of weight, and affecting (as we have often cause to suppose likely,) certain functions of the nervous system. However this be, I cannot doubt the reality of the fact as one of frequent occurrence. It is indeed merely an example, among so many others, of the partial subjection of the phenomena of life to the agents which are ever in movement around us."* 453.

The influence of weather in producing drowsiness is a subject of common observation. That effect is usually found, and has commonly been noted, when the air is moist and loaded with electricity. The same state of atmosphere, which seems to give birth to congestion about the head, disposes to what are called "bilious attacks"—conditions of the liver, in which congestion probably plays a principal part also. Subjects of this nature appear to us, as we have remarked, to receive less attention from medical men than they deserve. A right understanding of them may lead to the prevention of much annoyance, not to say serious disorders, and those who are subject to determination of blood to the head, would do well to devote some attention to them.

---

* "During the month preceding the time when I am now writing, (April, 1836,) there have been two or three very singular and sudden depressions of the barometer, (one of more than an inch within a few hours, from a point already low,) attended by gales of unwonted violence on the southern and western coasts of England. It has occurred to me on all these occasions, but particularly on the latter, to observe similar influence, as repects sleep, on myself and many different persons; taking for evidence those in ordinary health, rather than such as were suffering under malady at the time."

### On the Medical Treatment of Old Age.

This is really an important matter, however some may smile. To the patient it is important, for the nearer the old man approaches the grave, the more repugnant he usually is to step into it. To the medical attendant it is important also, for his *best* patient is not unfrequently his oldest one.

Nor is the medical treatment of old age so much a matter of moonshine as some persons may suppose. It has its peculiar diseases, its dangers to be warded off, its tendencies to be consulted, its weakness to be supported. To do all this requires discretion, experience, skill. The old practitioner knows this sufficiently, but the young one often does not. Dr. Holland justly observes :—it is understood that the rules which apply to other periods of life require alteration here, but the causes and extent of this are not always considered. True it is that old age is not to be reckoned merely by number of years. Family temperament, individual constitution, and the incidents of life, all concur to modify the time at which those changes begin which warrant the term in a physiological sense, and to effect no less the rate at which they proceed. Old age, moreover, may be said to be unequal in different individuals, as respects the different parts and functions of the body; —this diversity arising out of the same general causes which have just been assigned. Hereditary malady, or incidental disorders, often bring on premature decay in one organ, while others are comparatively untouched by time. In short, the treatment of old age requires much judgment and much attention.

Dr. Holland first touches on the changes which occur in the body in old age. We need not specify them. But we may allude to the decay of the mind.

"It is one opinion on this subject that the sensorial functions, later perhaps than others we possess in reaching their respective completeness in each individual, are the first which begin to decline. The assumption is a very doubtful one: but however this may be, the enfeeblement is certainly not in the same ratio as to time for all the faculties; the losses not equal of the ideas of knowledge of different classes, and different periods of acquisition. Does not the faculty of receiving and associating fresh impressions for the most part decline earlier, than the power of combining and using those formerly received? Is not the faculty of directing and fixing combinations or successions of ideas one of those earliest impaired? In a lady of 91, whom I am now attending, the decay of the mind shows itself especially in the dependence of the course of ideas on the sound of words. A word, or even part of a word, of double application, will suddenly, and without the consciousness of change, carry off the mind to a new and wholly foreign subject. In another elderly patient, whose memory is singularly tenacious as to persons and detached events of past life, there is a singular incapacity of associating them together by any reasonable link; and the slightest relations of time or place sufficient to carry the mind wholly astray from its subject. Such instances, by no means uncommon, have close kindred to the state of dreaming; the power of combining and directing the course of thought being similarly impaired in both cases." 273.

It is not wonderful that the memory of recent circumstances should be fleeting in those advanced in life. Ideas are impressions received through the medium of the senses—in old age, the senses are impaired, the impressions consequently less vivid—they are naturally therefore less permanent. This explains why the memory of events long passed should be more perfect than that of events which have recently occurred; but it does not explain why the former should be as perfect as it often is. Perhaps it may be that the circle of the octogenarian's ideas being bounded, the continual recurrence to the existing stock rivets them more strongly in the memory. We should say, however, that, as a general rule, the memory of the old is enfeebled every way; the oldest and most familiar objects are frequently forgotten, or only partially remembered. We quite agree with our author, that:—

"It would seem, as respects the decay of memory more especially, that the power of recollecting words and names is lost earlier and more readily than that of events, both in effect of old age, and of accident or disease. This fact is strikingly attested in paralysis and other cerebral disorders; where, the ideas still remaining distinct, words are partially or wholly wanting for their expression; or others, by a strange and inexplicable perversion, are substituted, having no relation to the intended sense.

In another chapter I have noticed (what perhaps is more hypothetical) the absolute tardiness of mental operations in old age, whether of perception, volition, memory, or thought. It seems impossible to exclude the direct notion of time from these cases, as compared with others where the functions are in full vigour of power and exercise." 274.

But to turn to more medical and less physiological matters. Dr. Holland lays it down as a canon, obvious when enunciated, too often forgotten or set aside, that we ought not to interfere,—or, if at all, with care and limitation,—in those cases where changes, irretrievable in their nature, have occurred in any organ or function of the body. How frequently do we see this truism practically denied. Hospital physicians and surgeons, more especially, acquiring, as they do, rough methods of treatment and a certain degree of hastiness of decision, are apt to discharge their drugs and instruments upon all symptoms and all ailments, and would seem to consider the removal of the complaint for which they are consulted the great and paramount and imperative object, even though the patient is removed along with it. Dr. Holland thus touches on the delicate considerations which cluster round this question.

"To urge medical treatment, in face of distinct proof to this effect, is to sacrifice at once the good faith and usefulness of the profession. This is a point the more needful to be kept in mind, as the patient himself, and those around him, are rarely able or willing to recognise it. It is often an exceedingly nice question of conscience, as well as of opinion, to define the extent to which practice may rightly proceed in such instances; always admitting, as must be done, that something is due to the feelings of the patient; something also to the uncertainty of our own judgments, antecedently to actual experience. This question in medical morals, like so many others, cannot be treated as a general principle only. The integrity and discretion of the practitioner must ever be appealed to for guidance in the endless variety of particular cases. In some, concession to a certain extent is safe, or even justified by indirect advantage to the patient. In others, mischief alone can arise from this meddling with the course of nature; and bad faith or bad judgment are involved in every such act of practice." 276.

There is always this drawback on negative practice in what we consider irremediable lesions, that our judgment is fallible, our means of determining what is and what is not curable imperfect. If too confident either way, we may inflict mischief—by leaning to the side of drugs and hope we may interfere injuriously—by a prognosis over gloomy we may withhold relief which it would be in our power to offer. This is only one of the thousand illustrations that present themselves of the impossibility of acting in the practice of medicine upon certain general rules, and of how much depends on the individual practitioner.

Our author proceeds to comment on the injudicious neglect which has been shown, in modern times, to the humoral pathology. Of this there cannot be a question. The changes of the solids have usurped our attention, monopolized our remedies, and the fluids have been treated with as little respect as ditch-water. But chemistry is rapidly undeceiving us, and a fair field lies open this way for research, and, probably, discovery.

Habitual discharges have, as a consequence of our outrageous solidism, been interfered with and suppressed, to a very mischievous extent. The evils of such practice tell most strongly on old age. A little violence, at that period, goes a great way in damaging the machine, and, no doubt, by such violence, it often is damaged. We seize the opportunity of drawing attention to the subject, and we dwell with pleasure on the judicious cautions of our author. He instances some of the particular discharges with which he counsels us not to meddle.

1. "In the Catarrhus Senilis, for instance, and the various bronchial affections of old persons, we have to consider whether it is expedient to put an active check upon them (therein complying with the demand almost always made from the physician), or whether the mucous or other discharges they involve are not really a safeguard to the constitution; an outlet provided for that which, retained in the blood, would create disorders of more critical kind? Without affirming it to be so in every case, my experience assures me that, in many, the practice of restraining such discharges is injurious in the attempt, hazardous if it be really effected. I entertain no doubt, from proofs furnished at every period of life, and particularly from the evidence by metastasis (of which I have noted many curious instances) that these large mucous secretions are often as essential to health as the secretions from other organs, which we assiduously seek to sustain. The necessity for them, having doubtless some relation yet unknown to the healthy properties of the blood, varies exceedingly in different habits, and at different ages; and this variation we can often distinctly connect with the state of other secretions; sanctioning the term vicarious, though not perhaps in the absolute sense in which it is sometimes used. We may seek to moderate occasional excess; to facilitate expectoration; and to regulate the cough so that it shall interfere as little as possible with rest. But to urge practice beyond this, in control of habitual symptoms, is more frequently a mischievous interference than a right direction of medical treatment.

The latter symptom, of cough, is that for which the physician's aid is most generally invoked, and where concession is too often and indiscriminately made. For, whether the secretions from these membranes be necessary or not, the act of coughing is at all events needful for their extrication or removal; and cases must have occurred to every practitioner where it is difficult to avoid the conviction that the sudden arrest of this, by opiates or otherwise, has accelerated a fatal event."

All this is very practical and very sound. It were well if it were more attended to. In the catarrhus senilis we have found most benefit from those sedatives which do not check secretion, digitalis and the prussic acid.

2. Many hesitate to interfere with cough, who do not hesitate to interfere with an attack of lithic acid gravel. Dr. Holland remarks:—

"A similar caution may be given as to interference with the secretion of lithic acid from the kidneys, generally increased in amount in old age. Alarm is often excited by the large quantity of this coming away, and alkalies are as largely given to obviate it. When there is tendency to concretion or occasional lodgment in the kidneys, this may be proper; but where the discharge is free and without distress, and there is no obvious disorder of internal organs attending it, the wisest practice is, generally, that which most refrains. This discharge indeed is often the most effectual outlet for that which, retained and accumulated, becomes a source of active disease, particularly to the brain, in those of advanced life. I have known many patients never well, unless when such removal of animal matters through the urine was largely going on." 278.

### On some Points in the Pathology of the Colon.

Dr. Holland doubts whether all the functions and disorders of this bowel have obtained proper attention in practice, or their influence in producing disorder elsewhere been sufficiently regarded. He deems it certain, as do we, that the colon is not simply an organ of transmission, but an organ of excretion in no unimportant degree. The diseases or disorders to which he refers are not those with which pathologists are familiar, but rather such as occur to the observation of physicians in practice, and possess, perhaps, some degree of vagueness.

"1. Many morbid states attributed, with the too frequent vagueness of medical language, to the stomach and liver, are chiefly, as I believe, connected with the colon as their seat and source. This remark applies alike to disorders of secretion, and to those in which the symptoms depend either on nervous sympathy, or simply on the mechanical effects of juxta-position and attachment of parts. As respects the first of these cases, it cannot be doubted that many of the various egesta from the bowels, usually termed bilious, and treated as such, have no other relation to bile than that of mere admixture. They are separated from the vessels or glands of the larger intestine by exudation or secretion,—act upon the contents of the bowel, as well as upon the living parts of the system, according as they deviate in quantity or quality from the healthy state,—and, in the excess of such deviation, indicate a state of the glands and secreting membranes, or it may be of the system at large, connected with some of the most important diseases of which we have knowledge." 171.

The "coffee-ground" matter, often described as disordered bile, is believed by Dr. Holland to be separated in great part in the lower intestines. We should say that the prevailing opinion refers such egesta to exuded blood, rather than to bile. Our author goes on to remark, that those secretions also which resemble chopped grass or spinach have probably the same origin; and even of the liquid which is called green bile it is doubtful what proportion may come from the liver. The colour of what passes from the bowels is often the effect of changes taking place within the intestine itself. This may readily be conceived, looking to the many materials present for chemical combination, both in the egesta and secreted fluids; and to the facility with which colour is changed from very slight causes, such as a small excess of acid, in matters thus composed. We feel some hesitation in subscribing to the opinion, that bile does not enter largely into the composition of the spinach or green stools. No doubt that vitiated intestinal secretions help liberally to compose them; but the colour seems to us to depend essentially upon bile. If Dr. Holland, however, is right, the liver is often charged with more than belongs to it, and mercurials are much abused.

2. " In like manner, those other secretions which have the character of minute specks of blood disseminated through a fluid; or sometimes of a black matter almost as deep as ink in colour; may be considered chiefly owing to exudation from the mucous membrane of the bowels. These secretions or exudations, which contain elements of the blood and partake much of its nature, may be extravasated, it would seem, from the membrane of any part of the intestines. But I have seen instances where products of this nature, coming away in large quantity before death, have been found on examination everywhere lining the coats of the colon, without any appearance of similar kind in the smaller bowels." 172.

The discharges in question vary in character from that of pure blood to that of the most inky fluid. Our author calls attention to the occasional relief which such evacuations afford. He has seen many cases of hypochondriasis of long duration, many also of protracted disturbance in the action of the heart, speedily and wholly relieved in this manner. Some of these excretions, he continues, are exceedingly noxious if retained in the body in which they are generated. In the case of a vigorous young man of two and twenty, the pulse, habitually about seventy, was brought down below forty, and rendered very irregular, by the passage through the intestine of a large quantity of that peculiar secretion resembling black oil; the pulse rising again immediately after it was removed from the body.

" The treatment of the cases mentioned above is not without many difficulties, and we have reason to believe that mischief is often done by a mistaken practice. To deal with these instances as with common diarrhœa, by opiates and astringents, may injuriously interfere with actions beneficial or needful in their result. On the other hand, irritation, by medicine or otherwise, to the vascular surface which is the source of these peculiar excretions, may inflict mischief of another kind, and equally prevent a salutary issue.

These difficulties, in truth, are common to cases of diarrhœa of more ordinary kind, arising out of the complication of the causes concerned;—the various ingesta received into the stomach,—the different secretions poured into the alimentary canal, as well from the large glandular apparatus of the liver and pancreas, as from the innumerable mucous follicles in every part of the canal,—and further, the great extent of vascular surface from which fluids may be separated by exudation or otherwise. In effect of this, a great variety of division and nomenclature has arisen as to these disorders; adding much to the embarrassment of the student, and increasing the chances of error in practice. It will probably be admitted by all who give honest expression to their experience, that even in these very familiar complaints the rule of treatment is less certain and consistent than in most other disorders, fluctuating perpetually between the doubt whether the diarrhœa present is to be checked by direct means, or allowed to proceed on its course?—whether medicines should be given with intent gradually to alter the secretions; or, with a bolder hand, to remove at once such as may be disordered or hurtful? This uncertainty of practice in bowel complaints, as they are termed, can only be obviated by experience, and the careful observation of what is mere mechanical irritation,—what vitiated secretion from other topical causes,—what the separation of noxious matters from the blood through the liver or intestinal membranes. Technical rules here may be too minute, as well as too general and vague, to be useful in practice. At all events, they can never supersede individual discrimination." 175.

We quite coincide with the preceding observations. If every man of experience were freely to admit the truth, he would probably confess that he had often experienced as much difficulty in treating diarrhœa, as in managing complaints esteemed of more consequence. The same doubts and the same difficulties apply to the treatment of other discharges, and for exactly the same reasons. Most discharges are salutary in some degree, and for a certain time—an evil, beyond a limit, difficult of determination. No general rules can assign the line of demarcation between the useful and the injurious, varying, as it does, in every case. The discrimination of the practitioner himself must determine it in each case.

3. Alterations of the calibre of the colon, from accumulations in it, or variations in the situation of its parts, may give rise to symptoms that demand consideration. The functions of the stomach, and even of the heart and lungs may be much disturbed by distention of the transverse arch of the colon, whether from air or from fæces. Dr. Holland is of opinion that many states of disorder supposed to belong to the stomach are really due to this cause. The distress so common to dyspeptic patients in the course of the night, and obviously connected with the bowels, seems frequently to belong to this part of them especially; the recumbent posture giving more effect to all such irritations as depend on its distention and pressure on adjoining parts.

"Disturbances in the colon are not unfrequently mistaken for complaints of the kidneys; and there is the greater liability to this mistake from the influence they really have, (either mechanically, or by some cause of sympathetic irritation, or by changes in the circulation), in disordering the state and secretion of the urinary organs. Whencesoever it arise, so close and frequent is this connexion, that we may always expediently begin the treatment of apparent disorder of the kidneys by full evacuation of the larger intestines; secure that we shall obtain alleviation in this way, if not entire relief. Few of the means especially directed to the urinary organs are so effectual as those which operate upon them through this part of the intestinal canal." 178.

We must confess that this does not square with our experience. We have not found that remedies acting on the colon exert the degree of influence attributed to them by our author. Attention to the bowels is requisite, no doubt, but it only acts in affections of the kidneys as an adjuvant to other and more powerful means. Such, at least, is the result of our experience.

"Many pains in the back and loins, which pass vaguely under the names of lumbago and rheumatism, are distinctly to be referred to the same cause. The effect of treatment here is usually the most certain proof; purgatives and injections relieving these symptoms speedily and effectually in some cases, failing in others. The very peculiar pains in the same parts, which attend the whole course of acute dysentery (and which are by no means sufficiently indicated in the common descriptions of this disorder), give further evidence how remarkably the morbid state of these bowels affects the adjoining textures." 178.

Cramps, he continues, and other spasmodic and painful affections of the lower limbs, are a frequent effect of the mechanical distention of different portions of this bowel; perhaps, also, of disordered secretions lodged within or passing through it. Of the latter we obtain proof in the very common concurrence of these symptoms with dysentery or ordinary diarrhœa. The acid also, which, according to recent observation, is for the most part predominant in the larger bowels, may be in such excess as to produce various disturbance by sympathetic irritation. The cæcum, of course, is most liable to distention, and the effects of this upon distant organs are so various and considerable as to require discrimination in practice. Dr. H. has seen more than one case, where pains were produced in the right leg, severe and constant enough to suggest the idea of more permanent disease in the joint or limb. We have seen a fixed and burning pain in the sole of one foot removed by evacuations from the bowels; and a fixed pain in the occiput was suddenly put to flight by the voiding of some hardened fæces.

"There is some difficulty in understanding the cause of those circumscribed swellings, to which all parts of the intestines seem liable, and which are sometimes so contracted in extent as to convey to examination the idea of hard, well-defined tumors, occasionally deceiving for a time by the resemblance. These swellings, as well as all other distensions and inequalities of the bowels, are more frequent in the female habit, and seem in certain cases connected with the peculiarities of the hysterical temperament. As the passage along the intestine, though impeded, is not generally closed in these cases, we must suppose that its coats are distended into a sac out of the direct course of the canal; originally, perhaps, from air generated suddenly or detained in this part of the intestine, or from the irritation of other more solid matters producing partial contractions, with relaxation of the intervening membrane and loss of power in the muscular coat. The sudden and unequal distension of the bowels from certain articles of food, or during the action of irritating medicines, illustrate the nature of these more circumscribed swellings. They are often relieved by a single active purgative (with which creosote or cajeput oil may beneficially be combined): but the habit of using frequent laxatives, by weakening the canal and rendering its motions irregular, increases the tendency to such disorders." 179.

In speaking of tympanitis, and of the various distressing sensations which arise from distention of the colon, our author takes occasion to add his quota of disapprobation of the system of violent, or, at all events, over-active purgation, which prevails too much both with patients and with doctors. It may be reasonably hoped that it is falling into sure but certain discredit with both.

Dr. Holland would direct particular attention to examination of the tongue. Yet he seems to own that he has not made much of it. He concludes with a hint, which may be acted on by our readers as an experiment:

"I will merely remark that, in many painful and spasmodic affections of this bowel, more aid might be got from external applications over the back, whether stimulating or anodyne, than is usually done. I have often found them greatly more effective here than on the abdomen, where they are generally used from regard to convenience; and this remark especially applies to the complaints of children. The remedy so employed, comes nearer in many places to the actual seat of disorder, and has more diffused effect on parts continuously affected. Where opium cannot be given internally, its employment in this way by embrocations is often very beneficial, provided these be adequately used. And even where such means are not effectual alone, they at all events come greatly in aid of those which are administered within." 182.

### ON HEREDITARY DISEASE.

Without entering into the philosophical and physiological inquiry which hereditary peculiarities suggest, we may say a word or two, and quote a fact or two in relation to them.

Hereditary tendencies to morbid actions play, probably, a more important part, and merit more the attention of practical men, than seems to be generally supposed. Dr. Holland thinks that the general law which regulates them, is that developed by Dr. Prichard—that all original or connate bodily peculiarities tend to become hereditary; while changes in the organic structure of the individual from external causes during life, end with him, and have no obvious influence on his progeny. Dr. Holland admits that this law has many exceptions, but to us it appears to be radically unsound. In the first place, it is clear that either Adam must have had a spice of most diseases in him, or else the subsequent origin of each must have been an infraction of the law. In the next place, everyday observation appears to contradict it flatly. Thus, a man of originally good constitution abuses it, and becomes phthisical. Will his offspring, begot while he labours under this disease, have a tendency to phthisis or not? Experience shews that they commonly will: and this in spite of the law. Take lues as another instance —take insanity as a third. Whichever way we look, we see reasons for doubting the existence of the law laid down by Dr. Prichard. That there is a limit to the transmission of morbid actions is unquestionable, but that limit appears to us more subtle and less defineable than the one assigned. One means of limitation undoubtedly resides in the influence of one parent neutralising more or less that of the other, in the constitution of the offspring. In families we constantly see a child present a resemblance in feature and in habit to one parent in particular. That parent may be the more healthy one or the less, and, in either case, the hereditary disposition obviously receives a bias. This infusion of fresh blood, acting in a manner which we do not comprehend, and which we cannot calculate, must interfere materially with the hereditary transmission of morbid peculiarities, and with the enunciation of any formula for their occurrence.

We may cite, for the purpose of graving the subject on the attention and memory of our readers, some instances of hereditary diseases which have occurred in the practice of our author.

"An instance is known to me of hydrocele occurring in three out of four successive generations, in one family,—the omission adding to the singularity of the fact, from its depending on a female being the third in the series, in whose son the complaint re-appeared. I am acquainted with a family, in which there are three examples, the father and two children, of inability to distinguish red as a colour. Another example, resembling the last, is known to me, where three brothers, and two or three children of their families, have the inability to distinguish between blue and pink. Instances of this kind as an hereditary defect are far from infrequent. I have known squinting to occur in every one of five children, where both parents had this defect. The repetition of cases of deaf and dumb children in the same family is well known to those who are concerned in institutions for the relief of this congenital defect. An example has recently occurred to me of that remarkable affection, the *suffusio dimidians*, existing in a father and his daughter; and brought on in each by circumstances singularly alike. In another family I have seen several instances of the peculiar tremor tendinum of the hands and arms, which is sometimes called the shaking palsy; and which here occurred in young persons of sixteen or eighteen, as well as in those more advanced in life. In this case, too, I had proof of the peculiarity having gone through at least three generations. Similarity in the form and setting of the teeth, as well as in the colour of the hair, is often observed between parents and children: and lefthandedness, from whatever this proceeds, sometimes takes the character of a family peculiarity. In a family where the father had a singular elongation of the upper eyelid, seven or eight children were born with the same deformity, two or three other children having it not. In like manner I have seen enlarged tonsils occurring in almost every individual of a numerous family, without other cause by which to explain it.

Eneuresis in children, from whatever source arising, occurs sometimes in so many individuals of the same family, as to make it almost certain that it has a common congenital origin. What is not less remarkable, as an instance of similar specialty, emphysema of the lungs has been ascertained to depend in the majority of cases on hereditary influence, independently of any disposition to tubercular pulmonary disease. Another instance which may be termed special, though belonging to a part of structure diffused over the whole body, is the hæmorrhagic diathesis." 21.

Dr. Holland concludes, and we see no reason to doubt it, that every organ and texture of the body is susceptible of deviations from the normal type or natural structure, capable of being conveyed to offspring; and of producing morbid actions, which are thus, under the name of disease, often propagated through successive generations. This very admission puts to flight the more restricted and hypothetical law of Dr. Prichard.

A curious form of hereditary morbid action is that which Duchesne and others have termed Atavism—where a bodily peculiarity, deformity or disease, existing in a family, is lost in one generation; re-appearing in that which follows. Speculation on its cause appears so unprofitable that we waive it. Indeed, while the ordinary process of generation continues a sealed book, it is not likely that deviations from the usual course of it would be otherwise than unintelligible.

"Connected with the foregoing is another curious variety in the transmission of disease, if so it may be termed, which has been little noticed by medical authors; the case, namely, of several children of a family being affected in common with some given malady, of which there has been no token on the side of either parent. An example has lately occurred to me in one family, of three sons and a daughter, every one of whom underwent an attack of hemiplegia before the age of forty-five, though neither father nor mother had been similarly affected. I find another instance in my notes, where three brothers severally suffered hemiplegia, and about the same period of life, without any record of the like event in the family. I have recently seen a fatal case of cerebral disease, with epileptic fits, in a young lady of twenty-four; two sisters of whom had died about the same age with similar symptoms, though neither parent had been subject to such disorder. I am acquainted with a family in which four children have died during infancy from affections of the brain, without any like instances in the family on either side. In another family, without any similar disease in the parents, three or four children had epileptic fits.

I have notes of several similar instances; chiefly, as I think, but not exclusively, disorders of the brain and nervous system. I have known three cases of diabetes mellitus in brothers, under ten years of age, in the same family; one of them fatal in result. In another instance, four cases of ascertained disease of the heart, all fatal about the same period of life, occurred to my notice in the brothers and sisters of one family; without any suspicion, as far as I could learn, of the parents having been the subjects of this disease. By a singular coincidence, another instance is known to me, where four brothers died, between sixty and sixty-five, of ossification and other disease of the heart; but here there appear to have been prior cases of the same kind in the family. In the instance of the deaf and dumb, already referred to, the examples are frequent and curious of several children being thus affected (five out of a family of eight, four from a family of seven,) without similar defect existing in the parents. At the School for the Deaf and Dumb in Manchester, in 1837, there were forty-eight children taken from seventeen families, the total number of children in these families being 106; and giving therefore an average of nearly three such cases in each family. Out of these instances, there appears but one in which the defect was known to exist in either parent; and we may rightly therefore consider this as one of the most striking examples of the fact under consideration.*

Some of these examples may perhaps be referred to the condition last mentioned, of the revival of an hereditary disorder, absent in one or more generations. But proof to this effect is wanting in other cases: and the remarkable fact remains, of several children of the same parents being affected in common with a given malady, of which there are no certain prior examples in either family. All physiology is at fault as to the solution of this phenomenon." 25.

It is possible that some of these may be instances of the revival of a hereditary disease, absent in one or more generations. But many cases will still be left, to which such a solution cannot apply.

A further variety in the hereditary transmission of disease is its limitation, more or less complete, to the males or females of the family affected. This fact is illustrated by two or three of the instances already given, and seems well attested in the case of the hæmorrhagic diathesis. What tends to involve the subject of the hereditary transmission of diseases in still greater obscurity, is the fact that exciting causes are usually requisite for their manifestation. This will go some way towards the explanation of the apparent influence of sex. The females of gouty family usually escape gout, probably because, in their cases, cold and excesses operate to a less extent.

On the whole, the subject deserves the attention of practitioners, nay deserves it more than would seem to be felt. How few make any particular inquiries on the tendencies to disease in the families of their patients. Yet such inquiries might often elicit valuable information, and afford a clue both to diagnosis and prognosis.

### On Medical Evidence.

We quite agree with Dr. Holland, that there can be few better tests of a sound understanding than the right estimation of medical evidence. It is a calculation of probabilities, and there are none who are so free from prejudice or so powerful of intellect, as to be able to single out each circumstance for consideration, and to give to it just its proper value.

Medicine can never be a demonstrative science, because given causes are

* "As a curious variation of the same general conditions, a case is known to me, where the children of parents, each deaf and dumb, are themselves free from that defect. In another large family I have known almost every child short-sighted, without either parent being so."

not followed by uniform effects. The wet stocking which gives one man rheumatism, gives another inflammation of the lungs, and another colic. The dose of calomel which stimulates A's liver and bowels, salivates B., and simply makes C. nervous. Men's constitutions differ so widely, that neither the causes of disease, nor its course, nor treatment, can be reduced to simple and unvarying expressions. Each case, consequently, is a special subject for study, and he is the best surgeon or physician who is able to study it best.

Yet these difficulties do not make medicine cease to be a science. On the contrary, it is a science of a high character, giving exercise to the strongest mind, and demanding the greatest powers of analysis. This very circumstance is unfortunate for physic. The demand on the intellect of its professors is too great. Indolence and ignorance both shirk the labour of investigation, and knavery affects it—all secure from exposure by reason of the incompetence of the public tribunal.

In the following observations we cordially coincide with our author:—

"It must be admitted, indeed, that this matter of medical testimony is too lightly weighed by physicians themselves. Else whence the so frequent descrip-tion of effects and cures by agents put only once or twice upon trial; and the ready or eager belief given by those, who on other subjects, and even on the closely related questions of physiology, would instantly feel the insufficient nature of the proof. Conclusions requiring for their authority a long average of cases, carefully selected, and freed from the many chances of error or ambi-guity, are often promulgated and received upon grounds barely sufficient to warrant a repetition of the trials which first suggested them. No science, un-happily, has abounded more in false statements and partial inferences; each usurping a place for the time in popular esteem; and each sanctioned by cre-dulity, even where most dangerous in application to practice. During the last twenty years, omitting all lesser instances, I have known the rise and decline of five or six fashions in medical doctrine or treatment; some of them affecting the name of systems, and all deriving too much support from credulity or other causes, even among medical men themselves." 3.

We fear that the cause of these delusions lies more deeply than a mere facility in admitting evidence. The merits of medical men and their rewards are adjudged by those who are ignorant of medicine, by a prejudiced and a credulous tribunal. How great must be the temptation to appeal to such prejudice, and to impose on such credulity. In the anxious competition, not merely for fame, but for subsistence, means will be resorted to, which, if not distinctly dishonest, tremble on the brink of dishonesty. Whatever speculation carries with it plausibility will be brought before the public, and supported by evidence not weighed in a very rigorous scale. This it is which makes us fear that medicine will be still, as it ever has been, oppressed with false theories and as false facts. Were the evil a mere error of rea-soning, a species of false philosophy, time, and the progress of human knowledge would assuredly remove it. But, being what it is, a mischief growing out of the constitution of society and founded on the necessities and vicious qualities of men, we look with despair on the prospect of a reformation.

But we proceed with an examination of what medical evidence *should* be. Dr. Holland observes:—

"Look at what is necessary, in strict reason, to attest the action and value of a new remedy or method of treament. The identity or exact relation of the cases in which it is employed,—a right estimate of the habits and temperament, moral as well as physical, of the subjects of experiment,—allowance for the many modifications depending on dose, combination, quality of the medicine, and time of use,—due observation of the indirect or secondary, as well as direct, effects,—and such observation applied, not to one organ or function alone, but to the many which constitute the material of life. All these things, and yet more, are essential to the completeness of the testimony. All can rarely, if ever, be reached; and hence the inevitable imperfections of medicine as a science. But more, doubtless, of truth and beneficial result might be attained, were these difficulties rightly appreciated, and the means of obviating them kept more constantly in view.

All this is very just. Yet who, at all conversant with medicine, sees facts so observed—observations so recorded—truth so sought in her most secret places? The very reverse of all this is witnessed. A few cases breed positive conclusions, and the hurry to observe is only surpassed by that to publish. The inherent difficulties which encumber Medicine are aggravated by the rubbish of fallacy and falsehood thrown upon her, and the wonder is that, amidst the confusion, so much is substantially correct.

The fallacies that beset all researches and all reasonings oppress us with additional force in physic—the same pernicious influence of words—the same tyranny of authority—the same prescriptive rights and vested interests in antiquity—the same meretricious blandishments of novelty—and we have, in addition, the ignorance and the interference of the public—an interference the more opinionated by reason of that ignorance. Our author observes only too justly:

"In no class of human events is the reasoning of '*post hoc, propter hoc,*' so commonly applied by the world at large, as in what relates to the symptoms and treatment of disease. In none is this judgment so frequently both erroneous and prejudicial. It would seem as if the very complexity of the conditions necessary to sound evidence tended to beget acquiescence in that which is lightest and most insufficient for truth. The difficulties occurring in practice from this source are great, and require a right temper as well as understanding to obviate them." 4.

It is quite clear that this is an evil the correction of which can come only from the profession. The public never will nor can be adequate judges of medical science. But in proportion as that is improved, the tone of professional reasoning will rise, and the weakness of the uninformed or the arts of the unprincipled among ourselves, will stand out in relief from the general tenor of medical conduct.

Thanks to morbid anatomy, medicine has already gained an immense addition of exactness—thanks to the numerical method, it is now in the act of gaining more. We agree with our author, that averages may in some sort be termed the mathematics of medical science. The principle is one singularly effectual in obviating the difficulties of evidence already noticed; and the success with which it has been employed of late by many eminent observers, affords assurance of the results that may hereafter be expected from this source.

He adds that:—In looking further to the chance of overcoming these difficulties in the future, regard must be had to the principle, now verified in so many cases, that in proportion to the complexity of phenomena, is augmented also the number of relations in which they may be surveyed and made the subjects of experiment. The application of this principle to medical science is every day becoming more apparent. Each new path of physical knowledge opened, each single fact discovered, has given guidance, more or less direct, towards the objects still unattained in physiology and the treatment of disease. The unexpectedness of some of the relations, thus determined and converted to use, is the best augury for further advances in this direction of pursuit.

On the one hand, then, we see morbid anatomy correcting the vagueness of symptoms, and assigning them to their organic causes—on the other hand, the numerical method tests the value of treatment. Here are two engines of modern improvement, working with a horse power which we cannot yet calculate. Each, especially the first, has effected much already. But morbid anatomy may be said to be yet unapplied to the fluids—and medical statistics are only in their infancy. Then chemistry is in our van. What combinations or what elements may it not produce or evolve for us—what may it not do for physiology and for therapeutics? He must be a timid man who does not think medicine still on the threshold of advancement.

That advancement may be opposed by two cardinal vices in medical men—excessive scepticism or extreme credulity—vices, we fear, of too prevalent a character, and which cannot be avoided with too much care.

On credulity we have already dwelt. But scepticism is almost as pernicious.

"Sometimes," says our author, "this occurs in effect of a temperament of mind (not uncommon among thinking men) which is disposed to see all things under doubt and distrust. There are other cases, where the same feeling, not originally present, grows upon the mind of physicians who have been too deeply immersed in the details of practice. The hurried passage from one patient to another precludes that close observation, which alone can justify, except under especial circumstances, the use of new remedies or active methods of treatment. From conscience, as well as convenience, they come to confine themselves to what is safe, or absolutely necessary; and thus is engendered by degrees a distrust of all that lies beyond this limit.

Though such scepticism be less dangerous than a rash and hasty belief, it is manifestly hurtful in practice, and an unjust estimate of medicine as a science; both as it now exists, and as it is capable of being extended and improved. No one can reasonably doubt that we have means in our hands admitting of being turned to large account of good or ill. Nor is it more reasonable to distrust the knowledge gained from a faithful experience as to the manner of using these, and others which may hereafter become known to us, safely and beneficially for the relief of disease. It is clear that between the two extremes just noticed, there is a middle course, which men of sense will perceive; and to which they alone can steadily adhere, amidst the many difficulties besetting at once the judgment and the conduct of the practitioner." 7.

This explanation of medical scepticism is a rational and just one. Nothing is more calculated to injure physic in the estimation of the world, than seeing its powers doubted and its science held cheap, by men who have reached to eminence in its practice. When the public remarks such scepticism in physicians like Radcliffe, Mead, and Baillie, they are naturally reminded of the joke of an Augur, that "he wondered how one could meet another in the street without laughing."

And yet such a low opinion of medicine as a practical art is decidedly unjust to it. Will any one contemplate the course of lues, of the phlegmasiæ, of intermittent fevers and other disorders, of many affections of the skin, of the majority of the complaints which come within the scope of surgery, and deny that medicine has great powers of cure? Will any one watch the neuroses and organic alterations of structure, and assert that it has not means of *alleviation?* Will any one study even fevers, and refuse to it the merit of guiding and directing to a safer issue than could in the gross be reached without it? We have a contempt for the mind of that man who really *knows* medicine, in its present state, and doubts its powers. In such a mind there must be something radically wrong—some obliquity which distorts the plainest ideas—some bias which warps judgment and turns comparison awry. That the earlier physicians should doubt physic is possible, without impugning their sense. It was then a tangled mass of hypothesis, to which morbid anatomy had given no solid bottom. But for men of the present day, if there be any, to harbour equal scepticism, is an ample qualification, in our opinion, for Bedlam.

Some persons appear to possess that strange constitution of mind, which in a given question knows no medium between universal affirmation or a total negation. Because there are many diseases uncontrollable by medicine, such persons protest there are none controllable by it. But the practice of physic is not a simple proposition, the affirmative of which is wholly true or false. It is a complex science, some parts of which are quite demonstrable, some probable, and some incapable of reduction to the formulæ of computation. Prejudiced minds of one kind will look to the one extreme, feeble or bigoted understandings of another sort will turn the other way. To avoid easy faith, to indulge in a just scepticism and nothing further, is the province of an intellect which cannot be created by canons, nor defined by language, and which is the mental constitution adapted to adorn and improve the science of medicine.

With the following short quotation we dismiss this subject—a subject of the greatest practical moment, not pertaining to the realms of abstraction and metaphysics, but coming home to the every-day business and the future advancement of our art. The remedy for the unhappy laxity of reasoning displayed by the bulk of the profession, can only be found in the extension of their general education, and in their attention being earnestly directed to questions of the present nature.

"I am the rather led," we quote our author, "to these remarks on the nature of medical evidence, and the causes moral as well as physical affecting it, looking at the wonderful advances which have been made in all branches of science; not by the addition of new facts only, but even yet more by new methods and instruments of research, and increasing exactness in every point of inquiry. The dissimilarity of the proofs, and the greater difficulty of their certain attainment, must ever keep practical medicine in the rear of other sciences. But its still wider scope of usefulness requires that the distance should be abridged as far as possible; and that no occasion should be lost, by improved methods as well as new facts, by more cautious observation, and greater exactness of testimony, of maintaining its fit place among the other great objects of human knowledge."

May his and our aspirations be realised.

### On Diet and Disorders of Digestion.

Dr. Holland would seem to take a gloomy view of our knowledge on the subject of indigestion. No doubt the complaint is troublesome—no doubt the opinions of authors are divided—no doubt there is much of inherent difficulty in the analysis of the phenomena of dyspepsia, and in the application of remedies to it—yet, notwithstanding these drawbacks, we conceive that much has been done to rationalise our views of the disorder, and that a great deal may be effected by experience, care, and judgment, in mitigation of its miseries.

Our author does not attempt to offer a systematic account of dyspepsia. He apologises for the desultory character of his remarks, by pointing to the similarly desultory nature of the complaint.

We should say that Dr. Holland's views lean to a rather dangerous latitudinarianism. He doubts whether rules of diet are not too strict—whether the bowels need be opened daily—whether regularity of hours is so indispensable. Such doubts may, perhaps, be reasonable, but they ought to be cautiously applied to practice, or, we feel confident, they will lead to disappointment. There appear to us to be two classes of cases of dyspepsia—those where the complaint is essentially seated in the apparatus of digestion itself, and the mind is affected through its medium—and those where the disorder is essentially mental, and the organs of digestion are affected secondarily. They run into one another, yet, practically speaking, the distinction holds, and remedies exert a very different influence on each. For the downright physical dyspepsia medicines are more requisite, and strict regimen more indispensable—for the mental, less rigid rules, more moral management, and a more dexterous application of means to circumstances are necessary.

Our author, however, suggests some remedial measures himself.

1. *Food.*—Dr. Holland enjoins, what certainly cannot be considered new, however true the precepts may be deemed:—first, that the stomach should never be filled to a sense of uneasy repletion. Secondly, that the rate of eating should always be slow enough to allow thorough mastication, and to obviate that uneasiness which follows food hastily swallowed. Thirdly, that there should be no urgent exercise, either of body or mind, immediately after a full meal.

Dr. Holland proceeds to remark that the great law of the living economy is this, that the appetite for food is lost or diminished, in exact relation to the impairment of the powers by which it is digested and converted to the uses of the system. And on this he founds a principle of treatment.

"I have mentioned," he says, "elsewhere the aids we are entitled to draw from the patient himself in this point of practice, where disease has restored nature for a while to its dominion over the appetites. It is rare that any mischief can arise from following this guidance, if care be taken to ascertain its reality. Nevertheless, the physician is liable here to various difficulties, created chiefly by the mistaken importunity of those around the patient. No conviction is harder to be removed than that which regards loss of appetite as itself the disorder,—food as the proper and certain remedy. Even the concurrence of the patient's feelings with the judgment of the physician is scarcely of avail against this persuasion. And when the former are altered in part by returning health, and old appetites and habits begin to recur, the risk of excess becomes greater, by the enfeeblement of that natural control which is the best security against it." 347.

However it may be in the phlegmasiæ, we have serious doubts of the propriety of Dr. Holland's advice in dyspepsia. So far as we have seen, the appetite in this complaint is peculiarly treacherous. It prompts continually both to a quantity and quality that prove injurious. Whoever has laboured under indigestion will, we think, admit this. Nothing is more common than for the dyspeptic to eat so much as to occasion most distressing fullness, and one of the hardest, yet most salutary lessons that he has to learn, is to control that very desire for quantity. On one point we are pretty certain to be right—in not forcing the appetite. We are not so sure of doing well in not controlling it. After all, the rule of practice, though difficult of definition, is tolerably easy of application. A trial or two will prove how far an appetite should be indulged, and individual cases become individual experiments.

We quite agree with our author, that active exercise should not be taken immediately before a meal, any more than shortly after it. He justly observes, that hard exercise and fatigue are often understood as a sanction for immediate and ample food, without regard to the expenditure of power that has taken place, or to the direction which the circulation has got towards the muscles and capillaries of the skin. Those who are exposed to the necessity of long and fatiguing journies speedily learn the error of this. But experience of such kind is generally needed to teach it; nor is this always sufficient against the force of early impressions, and the faulty habits of society.

He adds in a note:—

"I know from my own observation how well Government messengers, and the Tatar couriers of Turkey, have generally learnt this lesson, derived from the necessities of their manner of life, which are far more effectual for teaching than any other maxims. We apply the rule carefully and extensively in the management of our horses and dogs, without rightly heeding it for ourselves." 350.

And the same rule applies to active exertion of mind. Two great corporeal functions can scarcely go on amicably in full exercise at the same moment. The energies of the system, be the nervous or the vascular actions the efficient labourers, require to be concentrated on one important office to perform it well.

### 2. *Relations of Food and Sleep.*

"Setting aside," says our author, "the effects of particular articles of diet, which it would be impossible to specify, the practical questions chiefly regard the relation of time and quantity of food to sleep; and these are the fitter objects of study, as some averages are attainable and convertible to use. It is clear, for example, from observation both of man and other animals, that a certain quantity of food in the stomach, concurring especially with the habitual time of rest, tends to produce sound and healthy sleep;—that an excess in quantity brings on such as is broken, uneasy, and oppressed;—while sleeplessness is usually the effect of the stomach being empty and needing support. To these general facts, without inquiring into their physiological cause, may be referred most of the particular relations between sleep and food, and the precepts founded upon them. The fashions of society infringe largely upon the principles thus established; and though the powers of habit and accommodation in the system do much to lessen the evils that result, yet it is certain that some methods are greatly preferable to others, and that observation cannot better be employed than in ascertaining these.

In another chapter, on Sleep, mention will be made of the relation between the principal meal of the day and the time of rest. This is a question of constant occurrence in practice; and, without going into details, it is enough to say that much may be done for the restoration of sleep, where this is at fault, by altering the time of dinner to an earlier hour; so as not only to admit some bodily exercise in the interval, but also a slight supper before going to bed. The benefit of such change from the ordinary usage is often immediate; depending partly on the avoidance of the recumbent posture, and of efforts to sleep at a period of digestion unfit for this state; and further, and not less, on the direct effect of moderate repletion in disposing to rest." 352.

We must say that we hesitate in subscribing to Dr. Holland's opinions on this score. That a small quantity of aliment shortly before bed-time will occasionally conduce to sound sleep, we admit. But as a general rule, we are confident that two or three hours should elapse between the final meal and retirement to repose. Sleep so obtained will usually be more refreshing, and the morning's feelings and appetite much better than when a supper has been eaten. How frequently is benefit obtained from discontinuing suppers—how rarely do we find them agree. Habit, here, as elsewhere, occasionally steps in to supersede all general rules, and to interfere with prescriptions of common utility. But even habit, though too strong to be broken, is not too sturdy to bend, and a supper of beef and beer, or bread and cheese and ale, or of cigars and brandy and water, can be reduced to an innocuous minimum.

### 3. *Are Vegetables injurious in Dyspepsia?*

"Fashion still too largely tampers with the whole subject of dietetics; and injuriously, as regards the stability of its principles, and their application to practice. Of late years, for example, this fashion has directed itself against vegetable food;—an erroneous prejudice in many, perhaps in the majority of cases. Allowing, what is partly proved, that vegetable matters are carried indigested to a lower part of the alimentary canal than animal food, and admitting that more flatulence is usually produced from them, it still is the fact that a feeble digestion suffers no less, though it may be in different ways, from an exclusively animal diet. Morbid products are alike evolved; and some of these affecting not only the alimentary canal, but disturbing other organs and functions through changes produced in the blood.

I know the case of a gentleman, having the calculous diathesis strongly marked, in whom animal food, taken for three or four days, even in moderate quantity, invariably brings on discharge of lithic acid, as sand or gravel; suspended upon return to vegetable diet. This is a particular instance; but experience in gouty cases furnishes frequent and striking notices of the same general fact; thus indicating a large class of disorders, having much kindred with dyspepsia, in which excess in animal food rapidly becomes a source of mischief, not merely by overloading the alimentary canal, but by introducing morbid matters into the system at large. A persevering abstinence from any such excess may be reckoned among the most effectual preventives of gout in all its forms.

The rule of health being obviously that of blending the two kinds of food, I believe the exception more frequently required to be that of limiting the animal part in proportion to the other. The fashion of the day sets it down otherwise: and this is one of the subjects where loose or partial opinions easily get the force of precepts with the world at large." 354.

This question, like many others, can only breed endless difficulties and disputes, unless the parties engaged in it agree on the meaning of the terms they employ. If "dyspepsia" is to be taken in a comprehensive sense, to signify affections of the kidneys, and of the skin as well of the stomach and the bowels, then vegetables should not be generally condemned as articles of diet. But in the ordinary case of indigestion, accompanied as it is with oppression at the præcordia and flatulence, it is contrary to our experience, at all events, to assert that it is more frequently necessary to limit the animal part of the food than the vegetable.

No man in his senses orders a great increase of animal food because vegetables disagree. The diet quoad the quantum of meat remains much as before, nay is usually diminished, while the vegetable ingredients are curtailed. It would be difficult on theory to shew how this could harm the dyspeptic, and certainly, if our observation is worth any thing, it most commonly benefits him.

We have excluded from these remarks all cases save those of ordinary indigestion, as it usually presents itself in practice. In many instances of cutaneous eruptions, vesicular, pustular, and scaly,—in many gouty cases—in many where there is a disposition to the formation of an excess of lithic acid—animal food must be avoided or consumed in very moderate quantity. It is not our purpose to write a chapter on diet, and we confine ourselves to a brief remark or two suggested by our author's text, and as desultory as he confesses that to be.

4. *What are we to do with Wine?*—After some observations on this score, Dr. Holland adds:—

"As regards quantity of wine, for instance, the tests of what is to be deemed excess lie within the scope of ready remark, provided attention be fairly directed to the repetition of similar effects under circumstances reasonably alike. If the excitement of the spirits exceeds that of simple comfort (a condition not difficult to note), then it is certain that there is a state of brain, the frequent recurrence of which becomes a source of serious mischief both to body and mind. Or if, as happens in other constitutions, heaviness and drowsiness ensue speedily on the wine taken, equally is it certain that the quantity is in excess, and will be injurious in proportion to the frequency of repetition. Or if a hot dry skin, and increasing thirst, the inference is the same, and the result no less assured. Or, again, if the early hours of the morning are languid and oppressed, with headache of one kind or another, foul mouth, and weak or disordered stomach, the '*hesterna vitia*' may fairly be called to account, and wine probably as principal among these.

The familiarity of such tests makes them more valuable for practical enforcement; and there are few cases where the patient may not obtain from them, if fairly dealt with, a sufficient rule for his guidance. The same tests will apply to kind of wine as to quantity; but more ambiguously, from the many new conditions brought in, which neither chemical examination, nor common experience, has yet been able to explain. The importance of the question is fortunately much less than in the other case. It is true here, as with regard to solid food, that, if the quantity be duly limited, the kind, though by no means to be disregarded, becomes of much less significance. It may even be doubted whether any practical good has hitherto been gained by our researches into and classification of wines. The comparison of qualities, of the influence of which upon the body we are very slightly informed, gives a specious license to the indulgence in certain kinds, often quite as hurtful as the more careless use of which the preference of the moment forms the only rule." 358.

We may remark that such a test as this lies exposed to two objections. In the first place, by trusting to a patient's sensation of bare comfort, we use a test of most uncertain character—liable to vary with his fancies, or his frauds upon us. In the next place, the effects of habit must be taken into the account. Comfort on Monday results from one glass of wine—on that day month, the same amount of pleasurable sensation is only reached with the third or fourth glass. Yet no one surely will contend that what is best adapted to preserve health is so fluctuating a quantity.

5. *Bitters.*—" A point of consequence, also, is the manner of using bitters in the treatment of stomach disorders. Experience leads me to think that this is generally too large and indiscriminate. Even were our knowledge of their relative qualities more exact than it is, and our adaptation of them more just in consequence, we still might often render this action more beneficial in these complaints by lighter preparations than are usually employed. Bark, as having other and specific objects, may be excepted in part from the question. But there are various states of stomach in which the ordinary doses and strength of bitter infusions are injurious; while obvious good is got from a more moderate employment of the same means.

Frequently the best mode of using bitters in these cases is in direct combination with the aperient which may be necessary. Thereby a smaller quantity of the latter is usually rendered effectual; and the noxious effects of repetition materially abated. The addition of creosote to laxative medicines is beneficial in similar way; and for this, as well as other reasons, it deserves more trial in dyspeptic cases than it has yet obtained.

The theory and uses of tonic medicines—exceedingly vague, it must be owned, in every part of practice—are not least so in their relation to disorders of digestion. Of those fitly thus termed, iron, in one or other of its preparations, is, according to my experience, the most generally beneficial. Comparing it with the whole class of bitters, I doubt whether there is any one among the latter capable of being made so variously useful. Regard of course must be had, in employing it, to the cause and particular character of the dyspeptic symptoms. But the circumstances which create apprehension, often most needlessly, as to its use, may be readily obviated by its conjunction with whatever laxatives are required; and this, as in the instance of bitters, is often better done by direct combination than by separate employment. The ammoniated iron with an aloetic medicine, or the sulphate of iron in solution with the sulphate of magnesia or soda, will be found among the most beneficial of these forms; and applicable in numerous cases, without other difficulty than what may arise from the patient himself." 360.

We agree with our author that bitters are given too indiscriminately. It is not worth while running into exceptions and specifications, but we should say that these remedies must be given with a light hand. We cannot say that we have found quite so much occasion to be satisfied with iron as our author, but doubtless, with the precautions he observes, it is often serviceable.

### On Methods of Prescription.

It is impossible to become acquainted with many physicians or their prescriptions, without perceiving the frequent absence of a definite principle in their prescribing. A number of medicines are given at once, perhaps are combined together, because they have all been found serviceable in the complaint. Yet their action may be so various, that the one may neutralize the other, and it is obvious how vague, and perhaps erroneous, experience so obtained must be. There is this additional evil in it, that it tends to cripple our resources, for if the composite prescription disagrees or fails, we are ignorant which particular ingredient has done so, and we must either begin to experiment singly with each, which is a loss of time—or we have left ouselves without *matériel.*

"The best general rule seems to be, to define in every case, as far as can be done, the main object of present treatment,—to make this the basis of prescription,—and to admit, subordinately and under limitation, all such means as apply to collateral symptoms. This rule ought especially to be kept in mind, when a new medicine, or new applications of an old one, are on trial in our hands. Complexity here, especially that which arises from the admixture of new agents in the same prescription, disguises that which it imports us most to know, and which is always learnt with difficulty amid the many conditions tending to obscure the effect. In adding to these the uncertainty of combination, which is itself in most cases an experiment, we wrap one doubt within another, and play false with every just principle of medical research." 111.

From the treatment of disease the transition to its investigation is easy. If method, consistency, and principle were requisite in the one case, they are equally required in the other, and, too often, they are equally absent. Yet the analysis of disease, that is, the investigation of symptoms, must surely require both precision and system. From many phenomena the principal are to be distinguished, and disentangled of the encumbrances that hang upon it. Vague and inconsecutive questions, says our author, whether from timidity in the young practitioner, indifference in the older one, or irregular habits of thought in either, are not merely useless, but often positively hurtful. They defraud the physician of his own judgment, and put the mind of the patient into the state least apt to give right information. Rightly used, he continues, and not allowed to become too technical, a certain rule pursued in questioning the sick will strengthen, by disciplining, the powers of observation. Nor is there much danger lest this should cramp that higher and rarer quality (derived either from long experience, or from quick natural perception) which enables some men to comprehend at the first glance, to seize by a sort of intuition, all that is most needful to be noted in the diseases before them.

As a general rule, we should recommend the investigation of present symptoms before that of their history. We would also recommend ascertaining at once, which can generally be done, the organ mainly disordered. The questions relative to that should then be put. The examination of the other chief organs and functions must follow, each being separately investigated. The great point is to make the questions hang naturally together. In general, the plan is quite the reverse. The examination is straggling, desultory, inconclusive, and the impression conveyed to the listener, as well as to the examiner himself, is vague and unsatisfactory. But system, however perfect, must bend to circumstances, and there are times when some rambling question, or some gossiping remark, supplies a clue that method could not find. And with practice, what seems a circuitous proceeding, is made alike rapid and effective. As for intuitive diagnosis it is "bit or miss." We have never seen any pretender to it, who has not made the most grievous blunders, though, of course, he will now and then fire a happy shot.

The following cautions are judicious :—

"It is a frequent error in young practitioners to allow themselves to be betrayed into hasty diagnosis or prognosis of disease, either by their own nervousness, or by the importunity of the patient and those around him. The fault is serious ;—not merely as a source of embarrassment, but often by impairing integrity in practice, from the desire to redeem a wrong opinion thus given. It concerns greatly the reputation and usefulness of the profession that every caution should be exercised on this point. Forbearance in words, till the judgment is well decided, is therefore in some sort a duty, as well as an act of prudence. And it further behoves the physician to acquire as much mastery and readiness as possible in the observation of those symptoms which distinguish a disease or foretell its probable event,—another phrase, it may be, for medical experience ; yet with the advantage of being more explicit as to a very important part of practice. If mistakes in judgment be still made (and this will occur to every one), though their avowal is not always needful or desirable, yet I believe the character of the profession, as well as of the practitioner, to be best sustained by truth ; spoken as a man of sense and integrity will speak it." 113.

This latter recommendation is more praised than practised. We do not think the avowal of error generally advisable, unless the thing is too palpable to be either denied or concealed. The public are not judges of the difficulties of diagnosis, and will seldom do justice to erroneous opinions. The tribunal is consequently prejudiced, ignorant, and very frequently partial ; it may be proper to plead guilty to it, but few are so courageous as to do so.

Dr. Holland observes, that in the treatment of disease, it is not a bad rule to look first to the external remedies befitting each case, before determining those for internal use. This also may seem a trifling suggestion. Yet such technical arrangement, if involving no practical error, is generally useful, and especially to the young practitioner ; who is often painfully harassed, not only by the responsibility of instant judgment, but by the number of objects and methods present to him for consideration. Nor is it the technical aid alone which is of value here. It is well that every practitioner should keep in mind the expediency of attaining all that is possible by external remedies;—a discreet preference, strongly sanctioned by modern research, and in nowise incompatible with the bold and sufficient use of internal means, when called for by the more urgent necessities of practice.

And he adds:—The physician who leaves the bed-room of his patient, especially in cases of fever or acute disorder, without attending to more than the prescription of medicine and diet, has very imperfectly fulfilled his office. He is bound further to look to temperature and ventilation; the fit state of the patient's bed; his posture; the needful changes of clothing; the proper use of water for cleanliness or coolness; and the maintenance of quiet. These things, contributing alike to the comfort of the patient and to the chances of recovery from disease, are often, it must be allowed, passed over or too hastily dealt with, in the hurry of practice.

A Chapter is devoted to:—

### Points where a Patient may judge for Himself.

1. The patient may almost always safely choose a temperature for himself; and inconvenience in most cases, positive harm in many, will be the effect of opposing that which he desires. His feeling here is rarely, if ever, that of theory; though too often contradicted by what is merely such. This rule, continues Dr. Holland, may be taken as applicable to all fevers, even to those of the exanthematous kind; where, with an eruption on the skin, the balance between the outer and inner surfaces of the body, and the risk of repression might seem, and actually are, of greatest importance. In whatever stage the eruption be, if the patient expressly seeks for a cooler atmosphere, or cooling applications, they may be fully conceded to him, without fear of ill result; and under the guidance chiefly of his feelings as to the time during which their use may be continued.

We confess that we feel some doubts on this head. Take the case of erysipelas of the extremities. Cold is always, or almost always grateful—yet it is far from safe. Not that we would make a practice of disregarding the predilections of the patient with respect to temperature—far from it; we would only hesitate before we constituted his sensations the sole arbiter of its amount.

2. "In the majority of instances of actual illness, provided the real feelings of the patient can be ascertained, his desires as to food and drink may safely be complied with. Whatever be the physical causes of the relation (and they are yet beyond our research), the stomach itself is the best expounder of the general and more urgent wants of the system in this particular. But undoubtedly much care is needful that we be not deceived as to the state of the appetites, by what is merely habit or wrong impression on the part of the patient, or the effect of the solicitation of others. This class of sensations is much more nurtured out of the course of nature than are those which relate to the temperature of the body. The mind too becomes much more deeply engaged with them: and though in acute illness they are generally submitted again to the natural law, there are many lesser cases where enough remains of the leaven of habit to render every precaution needful. With such precautions, however, which every physician who can take schooling from experience will employ, the stomach of the patient becomes a valuable guide,—whether it dictate abstinence from or recurrence to food,—whether much or little in quantity,—whether what is solid or liquid, —whether much drink or little,—whether things warm or cold,—whether sweet, acid, or saline,—whether bland or stimulating to the taste." 78.

We own that we are rather startled at this passage. It seems to be an extensive letter of credit upon nature. It is rather strange that our stomach should play us so false in health, and turn to be so valuable a monitor, so excellent a physician, so oracular an expounder of the wants of the system in disease. We confess our scepticism, both on rational grounds and from experience. We have now and then seen a strange whim of the stomach prove serviceable, but we have more frequently seen it do mischief. Indeed the most difficult job of the physician is, in many instances, to control the stomach and its appetites, which could only be indulged with positive mischief. Dr. Holland, however, goes the whole length of his argument.

"It is not wholly paradoxical to say that we are authorised to give greatest heed to the stomach, when it suggests some seeming extravagance of diet. It may be that this is a mere depravation of the sense of taste; but frequently it expresses an actual need of the stomach, either in aid of its own functions; or indirectly, under the mysterious law just referred to, for the effecting of changes in the whole mass of blood. It is a good practical rule in such cases to withhold assent, till we find, after a certain lapse of time, that the same desire continues or strongly recurs; in which case it may generally be taken as an index of the fitness of the thing desired for the actual state of the organs. In the early stage of recovery from long gastric fevers, I recollect many curious instances of such contrariety to all rule being acquiesced in, with manifest good to the patient." 79.

Each man is guided by his own experience, and our's does not quite tally with our author's. No doubt that occasionally it is as he says; but sure we are that, in the great majority of instances, the stomach of the patient, or his expressed desire, whether the stomach play prompter or not, counsels strong food and drink and many heterogeneous things which the system would be all the worse for. This is more especially the case in convalescence. Few of us have not smarted from this very indiscretion on the stomach's part. It may be very true that our rules of diet are arbitrary, and that we often proscribe what might be useful in particular instances. But no one can deny that the articles our code permits are innocent, and that those it excludes are, to say the least, of dubious character. An impartial estimate will probably prefer a code of such a character, despotic though it possibly may be, to a laxity which is built on the whimsies of an invalid, and the capricious dictates of a falsely-educated stomach.

3. As regards, says our author, exertion of body, posture, continuance in bed or otherwise, the sick may generally be allowed their own judgment, provided it is seen to be one dependent on bodily feelings alone. And so equally with respect to fresh air, methods of exercise, and times of repose. In these things, as on points of diet, suggestions, founded on careful notice of the feelings of the patient, and watchfulness as to the effect of the first trials, are all that is required from the physician; and more than this often does mischief.

He goes on to say, that as respects mental exertion during illness or convalescence, much more caution is needful. Here the patient is usually less able to estimate his own power, and is more entirely at the discretion of those around him. The present condition of life among the higher classes produces as much of evil from excesses of moral and intellectual excitement, as from those of the stomach : and it is equally difficult to place watch and reasonable restraint upon them.

It appears to us that the mind and the stomach are pretty nearly in the same category, and that the patient is too often, in convalescence, led astray by the inclinations of both. In the middle and lower classes, however, the necessities and anxieties of life, the desire to retrieve lost time, and the apprehensions for a dependent family, are the hardest obstacles against which both patient and physician have to struggle. Mere inclination can generally be repressed by firmness, or, if indulged, it brings its punishment and is indulged no more. But what firmness can be exercised, or what punishment be effectual (unless indeed it be a final one) against the demands of positive necessity, felt with most especial hardship by many of the middle class ? These are some of the most trying scenes the physician has to witness.

4. The prudent physician, concludes Dr. Holland, without sacrificing his science or authority, may often draw knowledge from his patient as to the fitness of particular medicines, or the fitness of employing medicines at all. Frequently it happens that the latter, at some period or other of his disorder, usually when convalescence has begun, expresses strongly his feeling that medicine will do no further good, and that his future progress is best left to nature alone. Provided it be clearly seen that this does not arise from any whim or perverseness of mind (and the feeling may exist wholly without such), the medical man is bound to give attention to it, as suggestion at least for his further treatment. And if continued and repeated, at a time when the urgent symptoms of disorder are past, it may for the most part be rightly acquiesced in,—certainly so far as to make trial of the effects of the change which, being of a negative kind, can rarely produce much evil, even it fail of good.

Here we must take leave of Dr. Holland. It is impossible to lay down his book without an acquiescence in the decision of the public which has placed him in the first ranks amongst the practical physicians of the capital. Of urbane manners, with kindly feelings, observant at the bed-side, skilful in the use of remedies, and well-read in the medical literature of former days and of the present, Dr. Holland is well qualified for the position that he occupies, and is an example that young practitioners may safely imitate. Let us add, that we have every reason to believe that his mind is not warped by coterie feelings and class prejudices, that his bearing towards his brethren is fair and open, and that his candid mind, instructed by liberal reading and polished by society, is willing to allow their meed of merit to all, and does not confound competitors with rivals.

Of all that we have said the present work is the reflection, and none can peruse it without a large measure of instruction, and even more of respect for its author.

